# Retraction of: Integration of single cell multiomics data by deep transfer hypergraph neural network

**DOI:** 10.1093/bfgp/elaf024

**Published:** 2026-01-26

This is a retraction of Yulong Kan, Zhongxiao Zhang, Yingjie Wang, Yunjing Qi, Haoxin Chang, Weihao Wang, Zheng Zhang, Quanhong Liu, Xiaoran Shi, Integration of single cell multiomics data by deep transfer hypergraph neural network, *Briefings in Functional Genomics*, Volume 24, 2025, elaf009, https://doi.org/10.1093/bfgp/elaf009

The authors are retracting this paper following a post-publication review of the methodology used in constructing the hypergraph neural network. This review identified several critical issues that compromise the validity of the model and the reliability of the reported results.

Specifically:

• **Ambiguities in the high-dimensional embedding formula**, including an unclear definition of the embedding function and unreasonable parameter choices, which may have led to a poor-quality embedding space.

• **Errors in the definition and weighting of hyperedges**, notably an inaccurate formulation of neighborhood sets and failure to account for modality differences, potentially resulting in an unreliable hypergraph structure.

• **Logical flaws in the mathematical derivation of the propagation rule**, including incorrect definitions of normalization matrices and suboptimal strategies for initializing and updating weight matrices, which may have caused numerical instability and hindered effective learning.

Collectively, these issues undermine the reliability of the results and cast doubt on the conclusions of the research, so the authors and Editor-in Chief have decided to retract the article. All authors agree with the retraction.

